# MiR-449a suppresses the epithelial-mesenchymal transition and metastasis of hepatocellular carcinoma by multiple targets

**DOI:** 10.1186/s12885-015-1738-3

**Published:** 2015-10-15

**Authors:** Shu-peng Chen, Bao-xin Liu, Jie Xu, Xiao-feng Pei, Yi-ji Liao, Feng Yuan, Fang Zheng

**Affiliations:** 1 Guangdong Provincial Key Laboratory of Malignant Tumor Epigenetics and Gene Regulation, Medical Research Center, Sun Yat-Sen Memorial Hospital, Sun Yat-Sen University, No. 107, Yanjiang West Road, Guangzhou, 510120 China; 2 The State Key Laboratory of Oncology in South China, Cancer Center, Sun Yat-sen University, No. 651, Dongfeng Road East, Guangzhou, China; 3 Department of orthopedics, Guangzhou hospital of traditional Chinese medicine, No. 16, Zhuji Road, Guangzhou, China; 4 Department of Pathology, Guangdong Provincial People’s Hospital, No.107, Zhongshan Er Road, Guangzhou, China; 5 Department of Radiation Oncology, the Fifth Affiliated Hospital, Sun Yat-sen University, No. 57, Meihua East Road, Zhuhai, China; 6 Department of Breast Surgery, Hubei Provincial Cancer Hospital, No. 116, Zhuodaoquan South Road, Wuhan, China

**Keywords:** MiR-449a, Epithelial–mesenchymal transition, Metastasis, Hepatocellular carcinoma

## Abstract

**Background:**

Increasing evidence indicates that Epithelial–mesenchymal transition (EMT) can be regulated by microRNAs (miRNAs). MiR-449a is a liver abundant miRNA. However, the role of miR-449a in the metastasis of hepatocellular carcinoma (HCC) remains largely unknown.

**Methods:**

The expression levels of miR-449a were first examined in HCC cell lines and tumour tissues by real-time PCR. The *in vitro* and *in vivo* functional effect and underlying molecular mechanisms of miR-449a were examined further.

**Results:**

In the present study, we found that miR-449a was significantly decreased in HCC cells and tissues, especially in those with the portal vein tumor thrombus. In HCC cell lines, stable overexpression of miR-449a was sufficient to inhibit cell motility *in vitro*, and pulmonary metastasis *in vivo*. In addition, ectopic overexpression of miR-449a in HCC cells promoted the expression of epithelial markers and reduced the levels of mesenchymal markers. Further studies revealed that the reintroduction of miR-449a attenuated the downstream signaling of *Met*, and consequently reduced the accumulation of Snail in cell nucleus by targeting the 3’-untranslated regions (3’-UTR) of FOS and Met.

**Conclusions:**

Our data highlight an important role of miR-449a in the molecular etiology of HCC, and implicate the potential application of miR-449a in cancer therapy.

**Electronic supplementary material:**

The online version of this article (doi:10.1186/s12885-015-1738-3) contains supplementary material, which is available to authorized users.

## Background

It is known that invasion and metastasis, two of the most important hallmarks of malignant tumours, are the foremost fatal factors for human cancers. Identification of invasive and/or metastatic factors and an understanding of the underlying molecular mechanisms may provide novel targets for cancer therapy. Increasing evidence indicates that epithelial–mesenchymal transition (EMT) is a key event in tumor invasion and metastasis. During EMT, a morphological change from epithelial-like to mesenchymal-like appearance is accompanied by loss of cell-cell adhesion and activation of mesenchymal markers, such as N-cadherin, fibronectin and vimentin, as well as increased motility of tumor cells, which consequently facilitates tumor metastasis [1].

miRNAs provide functions essential for diverse biological processes by inducing translational inhibition and/or mRNA degradation of protein-coding genes [2, 3]. Deregulation of miRNA have been observed in various diseases, including cancer [4]. To date, more and more reports have indicated that a few miRNAs suppress (for example, the miR-200 family, miR-124, and miR-148a) [5–8] or promote (for example, miR-24 and miR-130b) [9, 10] EMT and tumor metastasis. Although some miRNAs (for example, miR-122, miR-26a, miR-331-3p and miR-216a/217) [11, 12] have been identified to regulate EMT in liver cancer, the role of miRNAs in the EMT of HCC deserved further investigation.

It has been reported that miR-449a is downregulated in multiple maglignancies. It inhibits growth of ovarian [13], endometrial [14] and prostate cancer cells [15], bladder cancer [16] and retinoblastoma [17], promotes apoptosis of colorectal and gastric adenocarcinoma cancer [18, 19], represses migration and invasion of non-small cell lung cancer [20], and also enhances the chemosensitivity to cisplatin in gastric cancer [21] and radiosentivity in lung adenocarcinoma [22]. The identified targets of miR-449a include HDAC [23], CDK6 and CDC25A [14, 24], BCL2 [18], cyclinD1 [21], E2F3 [25], C-MET [20], Notch1 and KLF4 [26] and androgen receptor [15]. These findings indicate that miR-449a may function as a potential tumor suppressor through diverse mechanisms. It was reported that the expression of miR-449a in HCC was inhibited by Histone deacetylases to tumorigenesis [27]. However, the role of miR-449a in the metastasis of hepatocellular carcinoma (HCC) remains largely unknown. In the present study, we showed that miR-449a displayed more pronounced reduction in HCC tissues with the portal vein tumor thrombus (PVTT). The restoration of miR-449a expression significantly repressed the *in vitro* migration and invasion, and *in vivo* pulmonary metastasis of hepatoma cells. Subsequent mechanism studies revealed that miR-449a attenuated EMT program by directly targeting FOS and Met. Moreover, the reintroduction of miR-449a attenuated the downstream signaling of Met, like activated phosphorylation of AKT-Ser473 and inhibitory phosphorylation of GSK-3α/β-Ser21/9, and consequently reduced the accumulation of Snail in cell nucleus, a transcription factor that promotes EMT. These findings provided novel mechanistic insights into the role of miR-449a in EMT and metastasis.

## Methods

### Tissue specimens and cell cultures

Normal liver tissues were collected from 18 patients who underwent resection of hepatic hemangiomas, and 66 HCC tissues were obtained from at the Cancer Center, Sun Yat-Sen University and Guangdong Provincial People’s Hospital (Guangzhou, China). All cases were histologically confirmed. None of the patients had received local or systemic anticancer treatment before the surgery. Written-informed consent was obtained from each patient, and the study was approved by the Institute Research Medical Ethics Committee of Sun Yat-Sen Memorial Hospital.

Four HCC cell lines (Hep3B, Bel-7402, SMMC-7721 and MHCC-LM9) were cultured in RPMI1640 medium with 10 % newborn calf serum. Another two HCC cell lines, Huh7 and HepG2, and normal hepatic cell line LO2, and 293FT, were maintained in Dulbecco’s modified Eagle’s medium supplemented with 10 % fetal bovine serum (FBS).

### RNA isolation and quantitative real-time PCR

Total RNA from cell lines and tissues was extracted with TRIzol reagent (Invitrogen, Carsbad, CA). cDNA was synthesized with the PrimeScript RT reagent Kit (Promega, Madison, WI). Real-time PCR was carried out using an ABI 7900HT Fast Real-time PCR system (Applied Biosystems, Foster City, CA) according to the manufacturer’s recommended conditions. The primer has been showed in Additional file 1: Table S1.

### Lentivirus production and HCC cell infection

Virus particles were harvested 48 h after pCDH-CMV-miR-449a-coGFP or pCDH-CMV-coGFP (System Biosciences, CA) transfection with the packaging plasmid pRSV/REV, pCMV/VSVG and pMDLG/pRRE into 293FT cells by using Lipofectamine 2000 reagent (Invitrogen). Lentivirus-miR-449a-coGFP and lentivirus-miR-ctr-coGFP were condensed and purified for 10^8^ MOI/200 μl. Next, LM9 and Huh-7 HCC cells were infected by lent-miR-449a and lent-miR-control, respectively, to construct the stable miR-449a-expressing and control HCC cells.

### Oligonucleotide transfection

MiR-449a inhibitor was synthesized by Genepharma (Shanghai, China). The sequence of siRNA FOS mRNA was 5’-CUGAGAAGCCAAGACUGAGUU-3’ (sense) and 5’-CUCAGUCUUGGCUUCUCAGUU-3’ (antisense). The sequences of the Met siRNA were 5**’**-GUCAUAGGAAGAGGGCAUUTT-3**’** (sense), and 5**’**-AAUGCC CUCUUCCUAUGACTT-3**’** (antisense), which were synthesized by Ribobo (Guangzhou, China). Oligonucleotide transfection was performed with Lipofectamine 2000 reagents (Invitrogen).

### Luciferase reporter assay

The putative miR-101 binding site at the 3’-UTR of *FOS* and *Met* mRNAs was cloned downstream of the cytomegalovirus (CMV) promoter in a pMIR-REPORT vector (Ambion). Two mutant constructs were generated by either deletion or mutations. The firefly luciferase construct was cotransfected with a control Renilla luciferase vector into LM9 cells in the presence of either lent-miR-449a or lent-miR-control. Dual luciferase assay (Promega) was performed 48 hours after transfection. The experiments were performed independently in triplicate.

### Colony formation assay

Twenty-four hours after infection, 200 or 500 infected cells were placed in a fresh six-well plate and maintained in RPMI1640 and Dulbecco’s modified Eagle’s medium containing 10 % FBS for 2 weeks. Colonies were fixed with methanol and stained with 0.1 % crystal violet in 20 % methanol for 15 min.

### Wound healing and invasion assays

Cell migration was assessed by measuring the movement of cells into a scraped, acellular area created by a 200-μl pipette tube, and the spread of wound closure was observed after 48 hours and photographed under a microscope. We measured the fraction of cell coverage across the line for migration rate. For invasion assays, 10^5^ cells were added to a Matrigel™ Invasion Chamber (BD Biosciences, Becton Dickson Labware, Flanklin Lakes, NJ) present in the insert of a 24 well culture plate. Fetal bovine serum was added to the lower chamber as a chemoattractant. After 48 hours, the non-invading cells were gently removed with a cotton swab. Invasive cells located on the lower side of the chamber were stained with crystal violet, air dried and photographed. For colorimetric assays, the inserts were treated with 150 μl 10 % acetic acid and the absorbance was measured at 560 nm using a spectrophotometer (Spectramax M5).

### Western blot analysis and immunofluorescence (IF)

Proteins were separated on SDS–PAGE and transferred to nitrocellulose membrane (Bio-Rad). The membrane was blocked with 5 % non-fat milk and incubated with the corresponding mouse anti-FOS, Met, E-cadherin, α-catenin, β-catenin, N-cadherin, fibronectin, vimentin (BD Biosciences, 1:1000 dilution), α-tubulin (Santa Cruz Biotechnology, Santa Cruz, CA, 1:1000 dilution), snail and GAPDH (Cell signaling Technology, Beverly, MA, 1:500 dilution) monoclonal antibodies. The proteins were detected with enhanced chemiluminescence reagents.

For the IF studies, cells were fixed with 4 % paraformaldehyde in phosphate-buffered saline and permeabilized with 0.2 % Triton X-100 in phosphate-buffered saline. Fixed cells were incubated with 1:2000 fluorescein isothiocyanate–conjugated phalloidin (Sigma, St. Louis, MO) or antibodies as indicated. Cells were counterstained with 4, 6-diamidino-2-phenylindole (DAPI) (Calbiochem, San Diego, CA) and imaged with a confocal laser-scanning microscope (Olympus FV1000, Tokyo, Japan).

### In vivo metastasis assay

MiR-control-Huh7 or miR-449a-Huh7 cells (2 × 10^6^) was suspended in 30 ml of PBS/Matrigel (1:1) and then implanted under the capsule of the left hepatic lobe of male BALB/c athymic nude mice at 4–5 weeks of age. The animals were killed and examined 38 days after tumor cell implantation. The liver and the lungs were removed and fixed with phosphate-buffered formalin. All the procedures are accordant with the Guide for the Care and Use of Laboratory Animals (NIH publications Nos. 80–23, revised 1996) and the Institutional Ethical Guidelines for Animal Experiments.

### Statistical analysis

Statistical analysis was performed using a SPSS software package (SPSS Standard version 16.0, SPSS Inc.). Differences between variables were assessed by the c2 test or Fisher’s exact test. For survival analysis, we analysed all patients with HCC by KaplaneMeier analysis. A log rank test was used to compare different survival curves. Multivariate survival analysis was performed on all parameters that were found to be significant in univariate analysis using the Cox regression model. Data derived from cell line experiments are presented as mean ± SE and assessed by a Two-tailed Student’s *t* test. *P* values of <0.05 were considered significant.

## Results

### Down-regulation of miR-449a is a frequent event in HCC patients with PVTT

**Fig. 1:**
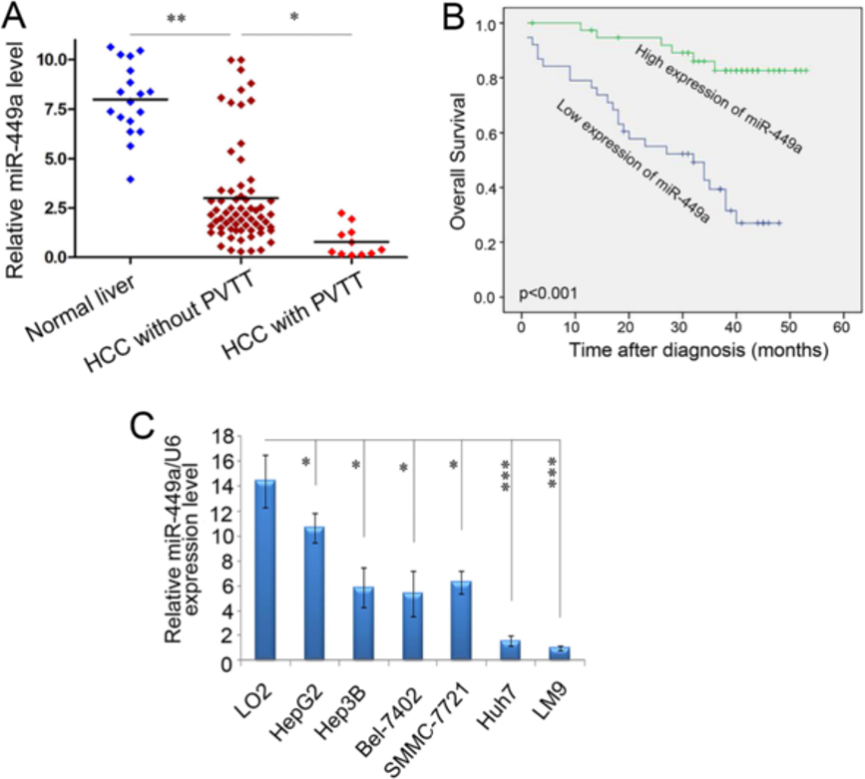
The levels of mature miR-449a expression in human HCC cell lines and tissues by real-time PCR. **a** miR-449a expression was reduced in HCC tissues with the PVTT. The levels of miR-449a in normal livers (*n* = 18), HCC tissues without the PVTT (*n* = 66) and with the PVTT (*n* = 11) were detected by qPCR and were then normalized to the expression of RNU6B to yield the relative miR-449a levels for individual sample. The median level of miR-449a in all HCC tissues was set as cut-off. Mann–Whitney U-test was applied to compare the differences in the miR-449a expression between the indicated cohorts. *P < 0.05, ***P < 0.001. **b** KaplaneMeier analysis for survival of patients with HCC as a function of miR-449a levels. Probability of patient survival, high-expression of miR-449a, *n* = 39; low-expression of miR-449a, *n* = 38 (*p* < 0.001). **c** Expression levels of miR-449a were examined by real-time PCR in LO2 cells and six HCC cell lines. Experiments were performed three times. Data are presented as means ± SE (*p* < 0.0001, independent t test)

To explore the roles of miR-449a in the development of HCC, the level of miR-449a was analyzed in 18 normal livers and 77 HCC tissues. Compared with normal tissues, the expression of miR-449a significantly decreased in HCCs. Further, those tumor tissues with the PVTT (11 samples) displayed more significantly reduction in miR-449a (Fig. 1a), suggesting that there was the potential correlation between miR-449a downregulation and HCC metastasis.

### Low-level expression of miR-449a is associated with an aggressive and/or poor prognostic phenotype of HCCs

To further investigate the clinicopathological and prognostic significance of miR-449a levels in patients with HCC, the levels of miR-449a in a large cohort of 77 HCC tissues were examined by real-time PCR. The median value of all 77 HCC samples was chosen as the cut-off point for separating tumours with low-level expression of miR-449a from high-level expression miR-449a tumours, thus 38/77 (49.4 %) HCCs had low-level expression of miR-449a, while 39/77 (50.6 %) HCCs had high-level expression of miR-449a (Table 1). Correlation analysis showed that low-level expression of miR-449a in HCCs was significantly associated with a more aggressive tumour phenotype (*p* < 0.05, Table 1). Kaplan-Meier analysis revealed that low-level expression of miR-449a was associated with short disease-free survival of patients with HCC (*p* < 0.001, Fig. 1b, Table 1). Further multivariate Cox regression analysis indicated that low-level expression of miR-449a is an independent prognostic factor for poor survival of patients with HCC (*p* < 0.001, Additional file 2: Table S2, Fig. 1b).

**Table 1:** Correlation of plasma miR-449a expression with patients’ clinicopathologic variables in human hepatocellular carcinomas

Variable	miR-449a			
	All cases	Low expression	High expression	*P* value^*^
Age (years)				0.710
≤50^a^	31	14 (45.2 %)	17 (54.8 %)	
>50	46	24 (52.2 %)	22 (47.8 %)	
Sex				0.768
Male	67	34 (50.7 %)	33 (49.3 %)	
Female	10	4 (40.0 %)	6 (60.0 %)	
Etiology				0.702
HBV	67	32 (47.8 %)	35 (52.2 %)	
None	10	6 (60.0 %)	4 (40.0 %)	
AFP (ng/ml)				0.052
≤20	44	17 (38.6 %)	27 (61.4 %)	
>20	33	21 (63.6 %)	12 (36.4 %)	
Liver cirrhosis				0.307
Yes	55	30 (54.5 %)	25 (45.5 %)	
No	22	8 (36.4 %)	14 (63.6 %)	
Tumor size (cm)				0.016
≤5	43	16 (37.2 %)	27 (62.8 %)	
>5	34	22 (64.7 %)	12 (35.3 %)	
Tumor multiplicity				0.014
Single	47	18 (38.3 %)	29 (61.7 %)	
Multiple	30	20 (66.7 %)	10 (33.3 %)	
Differentiation				
Well	10	3 (30.0 %)	7 (70.0 %)	0.137
Moderate	43	25 (58.1 %)	18 (41.9 %)	
Poor	20	7 (35.0 %)	13 (65.0 %)	
Undifferentiated	4	3 (75.0 %)	1 (25.0 %)	
Stage				
I	7	1 (14.3.0 %)	6 (85.7 %)	0.001
II	23	7 (30.4 %)	16 (69.6 %)	
III	31	15 (48.4 %)	16 (51.6 %)	
IV	16	15 (93.8 %)	1 (6.2 %)	
Distant metastasis				0.014
M1	9	8 (88.9 %)	1 (11.1 %)	
MX	68	30 (44.1 %)	38 (55.9 %)	

^*^Chi-square test; ^a^Mean age; HBV, hepatitis B virus; AFP indicates alpha-fetoprotein.

### Exogenetic overexpression of miR-449a suppresses HCC cell-line colony formation, migration and invasion in vitro and metastatic potential of HCC cell lines *in vivo*

To further investigate the effect of miR-449a on the metastasis of tumor cells, the expression levels of miR-449a were examined by real-time PCR in seven hepatocellular cell lines. The results showed that all six HCC cell lines (Hep3B, Bel-7402, SMMC-7721, MHCC-LM9, Huh7, HepG2) had lower levels of miR-449a expression than that in the normal hepatic cell line LO2. In addition, the metastatic HCC cell line LM9 showed the lowest levels of miR-449a (Fig. 1c).

**Fig. 2:**
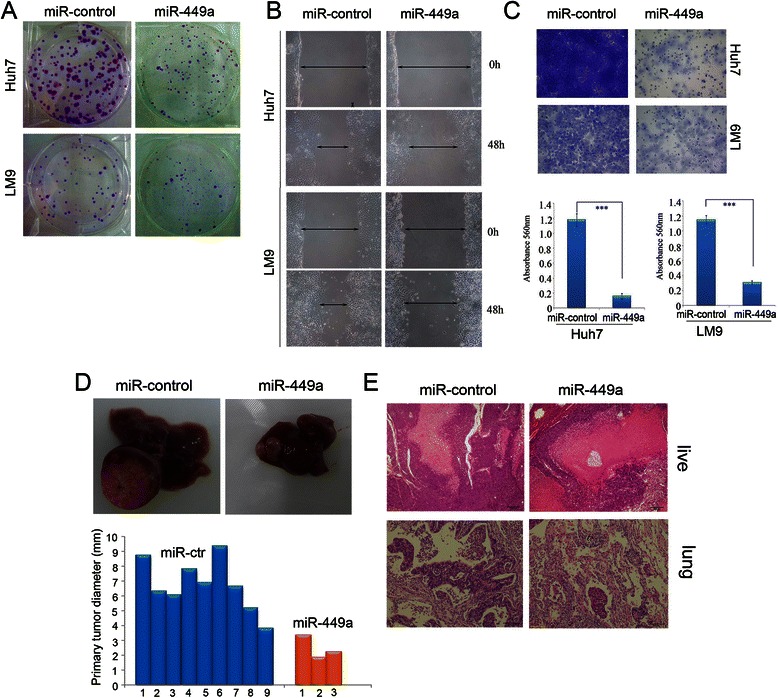
Exogenetic expression of miR-449a suppresses hepatocellular carcinoma cell invasion in vitro and reduces metastasis in vivo. **a** Effect of miR-449a on colony formation of the HCC cell line. Two hundred or 500 miR-124-infected Huh7 and LM9 cells were plated and a colony formation assay carried out. Representative results of colony formation of mock, miR-control-lentivirus-infected, miR-449a-lentivirus-infected Huh7 and LM9 cells. The results were reproducible in three independent experiments. **b** The wound-healing assay showed different cell motilities in miR-control-LM9, miR-449a-LM9, miR-control-Huh7 and miR-449a-Huh7 cells. The ectopic expression of miR-449a obviously inhibited the migration of LM9 and Huh7 cells. **c** Cell invasion was evaluated using a Matrigel invasion chamber. LM9 and Huh7 cells were infected by miR-control-lentivirus and miR-449a-lentivirus, respectively. All cells were subjected to a Matrigel invasion assay with fetal bovine serum as chemoattractant. Invasive cells were fixed and stained with crystal violet. The inserts were treated with 10 % acetic acid and the absorbance was measured. Both overexpression of miR-449a clearly inhibited the invasion of LM9 and Huh7 cells. Data are the means ± SD of three independent experiments. *p < 0.05, **p < 0.01. Scale bar: 100 mm. **d** Up, representative liver, treatments indicated. Down, the sizes of primary tumours in livers of mice, thirty-eight days after implantation of miR-control-Huh7 cells (average size, ± SE, 6.199 ± 1.63 mm) and miR-449a-Huh7 cells (average size, ± SE, 2.50 ± 0.79 mm). **e** The restoration of miR-449a inhibited the pulmonary metastases of HCC cells in vivo. miR-449a-Huh7 (Huh7 cells with stable expression of miR-449a) and miR-control-Huh7(control cells) were inoculated under the capsules of the left hepatic lobes of nude mice. Hematoxylin-eosin (HE) staining was performed on the serial sections of paraffin-embedded lung tissues (left and middle panels). Scale bar, ×100

The above observations prompted us to explore the potential biological function of miR-449a in HCC tumourigenesis and/or progression. Initially, the capacity of colony formation was evaluated in HCC cell lines (Huh7 and LM9) that were infected with miR-449a, miR-control. We found that miR-449a-infected cells had much fewer and smaller colonies than those in the miR-control-infected cells (Fig. 2a), indicating a growth-inhibitory role of miR-449a in HCC cells.

Next, the effect of miR-449a on invasive capacity of HCC cells was characterised by the wound-healing and Matrigel invasion assays. The results showed that exogenetic overexpression of miR-449a caused a suppression of cell migration in the LM9 and Huh7 cell lines using a wound-healing assay (Fig. 2b). Matrigel invasion assays demonstrated that exogenetic overexpression of miR-449a markedly reduced invasiveness of LM9 and Huh7 cell lines (Fig. 2c, *p* < 0.05).

To confirm the in vivo metastasis-inhibitive function of miR-449a, an orthotopic HCC mouse model was employed. Huh7 cell that stably expressed miR-449a (miR-449a-Huh7) and its control line (miR-control-Huh7) were, respectively, implanted under liver capsule. Thirty-eight days after implantation, cell growth in the liver and the lung was assessed. First, the size of the primary tumour nodules in the liver was examined. Tumor incidence was 56.25 % (9/16) in the miR-control-Huh7-injected mice and 18.75 % (3/16) in the miR-449a-Huh7 group. And we found that the tumour sizes (tumour diameters) were significantly decreased in the miR-449a group compared with that in the miR-control group (*p* < 0.01, Fig. 2d). To explore the role of miR-449a in tumour metastasis, we examined the number and the size of the tumor metastatic nodules under a microscope in the liver and in the lung. As shown in Fig. 2e, the number of pulmonary metastatic nodules was clearly decreased in the miR-449a group compared with that in miR-control group (9/16 vs 3/16 mice, *p* = 0.016, Fig. 2e, Table 2), but comparable rate of intrahepatic metastasis (3/16 vs 3/16 mice, *p* = 0.93, Fig. 2e). Collectively, these results indicate that miR-449a possesses metastasis-suppressive activity and its downregulation may facilitate the metastasis of HCC.

**Table 2:** miR-449a inhibited pulmonary metastasis

Groups	Metastasis		*P* values
	-	+	
Huh7-miR-control	7	9	0.016
Huh7-miR-449a	13	3	

The difference in the pulmonary metastatic rates between the miR-control-Huh7 and miR-449a-Huh7 was analyzed by Fisher’s exact test

### Silencing endogenous miR-449a promotes cell motility and induces the EMT phenotype

**Fig. 3:**
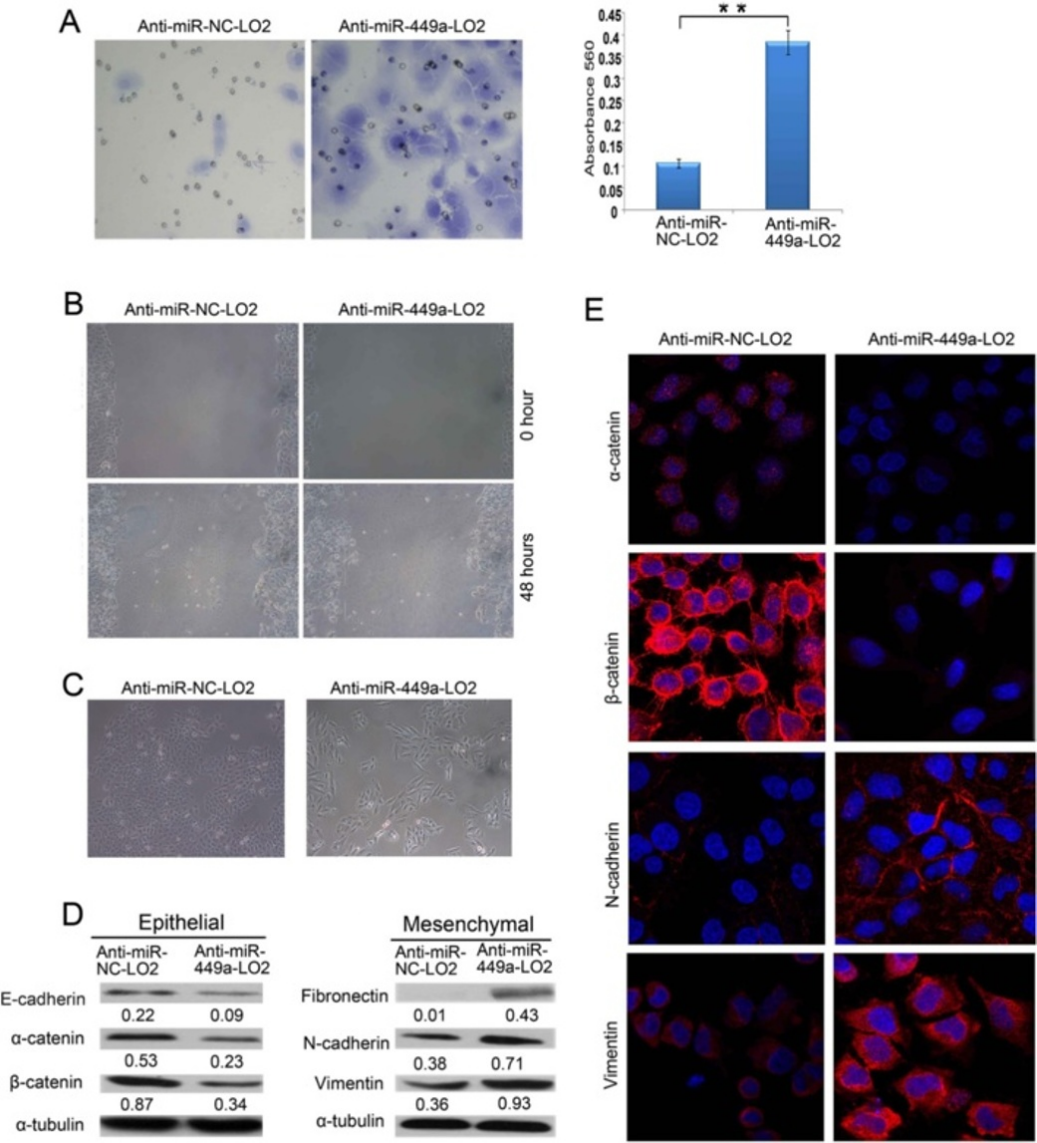
Silencing endogenous miR-449a promotes cell motility and induces the EMT phenotype. **a** The invasive properties of the LO2 cells transfected with between anti-miR-NC and anti-miR-449a were analyzed by an invasion assay using a Matrigel^™^ Invasion Chamber. Migrated cells were plotted as the average number of cells per field of view from 3 different experiments, as described in Methods. **b** Anti-miR-449a- LO2-transfected cells showed higher motility in a wound-healing assay. 48 hours posttreatment. **c** Cell morphyology of anti-miR-NC- LO2 and anti-miR-449a- LO2 cells. **d** Expressions of epithelial markers α-catenin, β-catenin and mesenchymal markers fibronectin, N-cadherin and vimentin were compared by western blot analysis between anti-miR-NC-LO2 and anti-miR-449a- LO2 cells. α-tubulin was used as a loading control. **e** IF was used to compare expression level/pattern of epithelial markers and mesenchymal markers between anti-miR-NC-LO2 and anti-miR-449a-LO2 cells. Epithelial markers α-catenin, β-catenin (red signal) were downregulated in anti-miR-449a cells; mesenchymal markers fibronectin, N-cadherin and vimentin (red cytoplastic signal) were upregulated in anti-miR-449a cells

Because cell motility is an important factor regulating cancer invasion and metastasis, the effect of miR-449a on cell motility was characterized by Matrigel invasion and wound healing assays. At first, because LO2 cell endogenously expresses the high miR-449a by the real-time PCR (Fig. 1c), we transfected LO2 cells with the anti-miR-449a and examined the cell motility. The Matrigel invasion assay showed that the invasiveness of the anti-miR-449a-LO2 cells was significantly higher than anti-miR-NC-LO2 cells (*P* < 0.001, Fig. 3a). Similarly, the wound-healing assay showed that cell migration at the edge of exposed regions was remarkably faster in anti-miR-449a-transfected cells than in anti-miR-NC cells (Fig. 3b). The data indicated that anti-miR-449a enhanced the cell motility.

And we found that the anti-miR-NC-LO2 cells maintained highly organized cell-cell adhesion. However, when plated at the same cell density, anti-miR-449a-LO2 cells exhibited a cell scattering phenomenon and loss of cell-cell contact, accompanied by the spindle-shaped, fibroblastic morphology (Fig. 3c). The morphological changes indicated that these cells may undergo EMT. To further demonstrate this phenotype, the effect of miR-449a on EMT was investigated by western blot and IF. In anti-miR-449a-LO2 cells, expression of E-cadherin, α-catenin and β-catenin decreased. On the other hand, all mesenchymal markers tested, including fibronectin, N-cadherin, vimentin, were elevated in anti-miR-449a-LO2 cells (Fig. 3d). These data reinforced that miR-449a overexpression may inhibit EMT. The Western blot results were confirmed by IF analysis (Fig. 3e). In particular, the mesenchymal marker fibronectin was completely undetectable in LO2-anti-miR-NC cells, whereas anti-miR-449a-LO2 cells showed positive staining of fibronectin in the cytoplasm. In addition, vimentin intermediate filaments localized in a concentrated and polarized pattern in anti-miR-NC-LO2 cells; however, in anti-miR-449a-LO2 cells a network of vimentin intermediate filaments was clearly visible. Taken together, these results strongly suggested that silencing endogenous miR-449a induces the EMT phenotype.

### Overexpression of miR-449a inhibits HCC cell and *in vivo* EMT

**Fig. 4:**
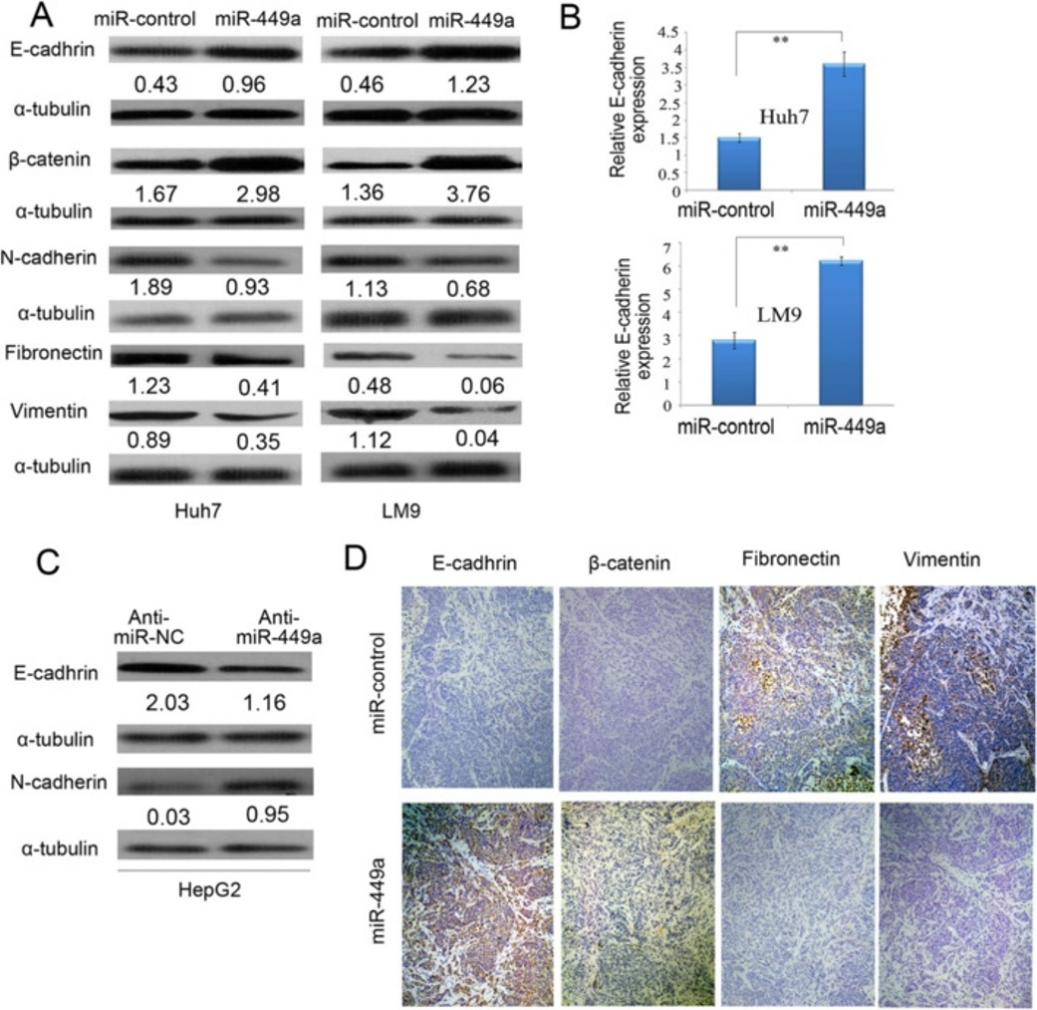
Overexpression of miR-449a in Huh7 and LM9 cells reverses epithelial mesenchymal transfer. **a** Expression of epithelial markers and mesenchymal markers were compared by western blot analysis between miR-control-Huh7, miR-control-LM9 and miR-449a-Huh7, miR-449a-LM9 cells. α-tubulin was used as a loading control. **b** miR-449a overexpression enhanced the mRNA level of E-cadherin in hepatoma cells. **P < 0.01. **c** The antagonism of endogenous miR-449a promoted the EMT of hepatoma cells. HepG2 cells were transfected with anti-miR-449a or its control (anti-miR-NC) for 72 h and analyzed by immunoblotting. α-tubulin was used as a loading control. **d** Immunohistochemistry staining showed a decreased expression of fibronectin and vimentin and an increased expression of E-cadherin and β-catenin in tumour tissues originating from miR-449a-Huh7 cells, compared with that originating from miR-control-Huh7 cells. Scale bar: 50 mm

Since EMT is well known to be involved in invasion and metastasis of cancer cells, we asked whether or not the levels of miR-449a in HCC cells can reverse the EMT induction. We assessed the epithelial and mesenchymal markers by western blot in miR-control and miR-449a-overexpressing LM9 and Huh7 cells. The expression levels of three epithelial markers (E-cadherin, a-catenin and b-catenin) increased (Fig. 4a), while the levels of three mesenchymal markers (fibronectin, N-cadherin and vimentin) decreased. On the other hand, loss-of-function analyses in HepG2 cells showed that the inhibition of miR-449a with antimiR-449a reduced E-cadherin expression and elevated N-cadherin level (Fig. 4b and c). These results indicated that overexpression of miR-449a represses the EMT phenotype of Huh7 and LM9 cells.

Furthermore, IHC staining demonstrated that the tumors in the livers of mice originating from miR-449a-Huh7 cells had decreased expression of fibronectin, N-cadherin and vimentin and increased expression of E-cadherin, α-catenin and β-catenin, compared with that from miR-control-Huh7 cells (Fig. 4d). These data showed that overexpression of miR-449a inhibited the EMT of Huh7 cells both *in vitro* and *in vivo*.

### MiR-449a directly targets FOS and Met 3’-UTR

**Fig. 5:**
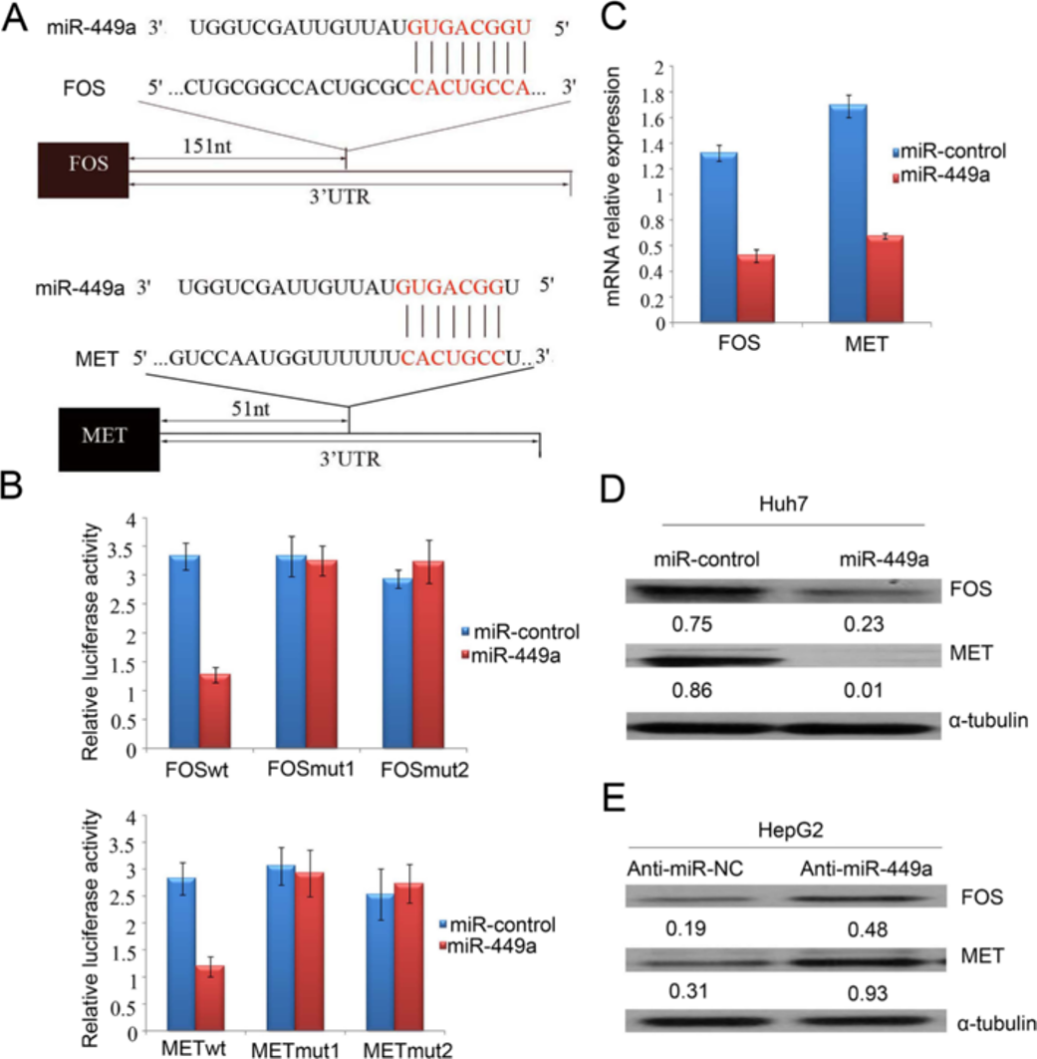
FOS and MET are targets of miR-449a. **a** Schematic illustration of the predicted miR-449a-binding sites in FOS and MET 3’-UTR. **b** FOS and MET were targets of miR-449a. MiR report constructs, containing a wildtype and two mutated FOS and MET 3’-UTR, were co-transfected into LM9 cells which were infected by miRcontrol-lentivirus or miR-449a-lentivirus. Relative repression of firefly luciferase expression was standardised to a transfection control. Data of the reporter assays are the means ± SE of three independent experiments. **c** mRNA levels of FOS and MET after miR-449a-induced expression in LM9 cells examined by real-time PCR. **d** Ectopic expression of miR-449a decreased endogenous levels of FOS and MET protein in LM9 cells. LM9 cells were infected with either lentivirus-miR-control or lentivirus-miR-449a for 72 h. FOS and MET expression was assessed by western blot. **e** The antagonism of endogenous miR-449a increased FOS and MET expression in the HepG2 cell. Anti-miR-NC or anti-miR- 449a was transfected into HepG2 cells for 72 h and analyzed by immunoblotting. α-tubulin was used as a loading control

We next explored the molecular mechanisms responsible for the EMT and metastasis-suppressive effect of miR-449a. Putative miR-449a targets were predicted using target prediction programs, miRanda and TargetScan. Our analysis revealed that FOS and Met were two potential targets of miR-449a. The 3’-UTR of FOS and Met mRNA contains a complementarysite for the seed region of miR-449a (Fig. 5a). To verify whether or not FOS and Met are direct targets of miR-449a, FOS and Met 3’-UTRs (Fig. 5a) and two mutants containing the miR-449a binding sites were cloned downstream of the luciferase open reading frame. These reporter constructs were used to cotransfect HCC LM9 cells. Increased expression of miR-449a upon infection, significantly affected the luciferase expression, measured by the luciferase activity. Conversely, when we performed luciferase assays using a plasmid harbouring the 3’-UTR of FOS and Met mRNAs, in which the binding sites for miR-449a were inactivated by site-directed mutant genesis, the luciferase activity of mutant reporters were unaffected by the simultaneous infection of miR-449a (Fig. 5b).

To determine if miR-449a affects FOS and Met expression in the HCC intracellular environment, we analysed the changes of FOS and Met expression in HCC cell line LM9 after miR-449a overexpression. Using real-time-PCR, we found that the mRNA levels of both FOS and Met were dramatically reduced in miR-449a-Huh7 cells, as compared with that in miR-control-Huh7 cells (Fig. 5c). Meanwhile, the protein levels of FOS and Met were also substantially decreased after ectopic overexpression of miR-449a in Huh7 cell lines as evidenced by western blot assays (Fig. 5d). On the other hand, knock down of miR-449a by 20-O-me-anti-miR-449a in HepG2 cells increased protein levels of FOS and Met (Fig. 5e).Taken together, these data support the bioinformatics predictions indicating FOS and Met 3’-UTRs as direct targets of miR-449a.

To determine whether or not FOS and Met are involved in miR-449a-inhibited invasion and migration, we first transfected Huh7 and LM9 cells with siFOS or siMet. The results showed that knockdown of FOS and Met by siRNA suppressed the invasive ability of Huh7 and LM9 cells (Additional file 3: Figure S1). Additionally, introduction of miR-449a had a greater inhibitory effect on cell migration and invasion than that of knockdown of FOS and Met alone. So these data indicate that FOS and Met are involved in miR-449a-inhibited invasion and migration. At the same time, we also confirmed that Notch1 is the another target of miR-449a in HCC (Additional file 4: Figure S2).

### MiR-449a levels are inversely correlated with mRNA expression of FOS and Met in HCC tissues

We further examined the mRNA expression of FOS and Met in 77 cases of HCC tissues. A significant inverse correlation between the levels of miR-449a and mRNA expression of FOS and Met was evaluated in our HCC cohorts. And low levels of miR-449a were more likely to be observed in HCCs with high expression of FOS or Met mRNA (*P* < 0.01, Additional file 5: Table S3).

### MiR-449a represses Snail signaling and its nuclear accumulation by directly targeting FOS and Met

**Fig. 6:**
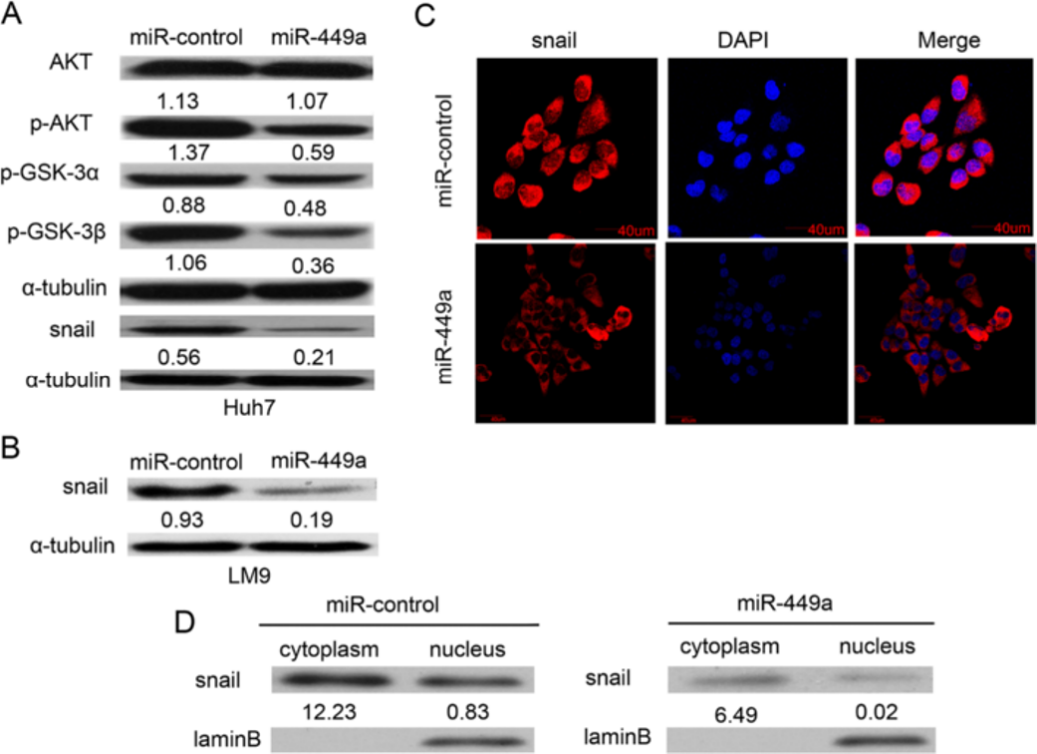
MiR-449a regulates AKT/GSK-3α/GSK-3β/Snail signaling and subcellular location of Snail. **a** miR-449a inhibited AKT/GSK-3α/GSK-3β/Snail signaling in Huh7 cells. **b** miR-449a repressed the expression of Snail in LM9 cells. **c** miR-449a reduced the nuclear accumulation of Snail in LM9 cells. miR-control-Huh7, miR-control-LM9 and miR-449a-Huh7, miR-449a-LM9 cells were analyzed by immunoblotting and immunofluorescent staining. Scale bar, 40 μm. **d** Expression of snail was compared by western blot analysis nuclear and cytoplasmic extraction of miR-control-Huh7 and miR-449a-Huh7 cells. LaminB was used as a loading control

It is known that the resultant nuclear accumulation of Snail transactivates the expression of mesenchymal markers and inhibits the transcription of E-cadherin [28]. And Met signaling activates AKT and abrogates GSK-3α/β activity, which in turn causes the reduced phosphorylation and ubiquitination of Snail [29]. Therefore, we investigated whether miR-449a repressed AKT/GSK-3α/β/Snail signaling cascades. The overexpression of miR-449a attenuated the activated phosphorylation of AKT-Ser473 and the inhibitory phosphorylation of GSK-3α/β-Ser21/9, and consequently reduced the expression level (Fig. 6a and b) and nuclear translocation of Snail (Fig. 6c). In order to explore sequestering of snail into cytoplasm, we performed nuclear and cytoplasmic extraction of cells after the overexpression of miR-449a. Western blot analysis further confirmed that the transfection with miR-449a had inhibited the nuclear accumulation of snail (Fig. 6d).

## Discussion

Although deregulation of miRNAs has been observed in various types of human cancer, the molecular mechanisms by which miRNAs modulate the process of carcinogenesis are still unclear. In this study, we observed that downregulation of miR-449a is a frequent event in HCC tissues, especially in those with the PVTT. Low-level expression of miR-449a was significantly associated with a more aggressive tumour phenotype and was shown to be a strong and an independent predictor of short disease-free survival for patients with HCC. In functional studies, reintroduction of miR-449a dramatically repressed HCC cell colony formation, migration and invasion *in vitro* and tumour metastasis *in vivo*. These findings suggest that miR-449a plays a critical role in the invasive and/or metastatic potential of HCC. The documents also reported that the miR-34 that shares the same seed sequence with miR-449 is tumor-suppressive and methylated in lung cancer [30]. And the function of miR-449 in cancer is further supported by experiments in several cancer cell lines in which miR-449 induces G1 arrest, apoptosis, and senescence by regulation of a series of key factors in cell cycle and apoptosis [16, 18, 31, 32]. These results indicate that miR-449a regulates tumor growth as a tumor suppressor, which could partially explain the correlation between low expression of miR-449a and poor prognosis.

The distinguishing feature of metastasis and invasive growth is the transition of tumour cells from an epithelial to a mesenchymal morphology, known as the epithelial–mesenchymal transition (EMT). EMT who is characterized by the decrease of epithelial marker, increase of mesenchymal markers, change of morphology, loss of cellular adhesion and enhancement of cell motility appears to be a key event in tumor invasion and metastasis [33]. The expression of EMT-associated markers are been regulated by multiple transcription factors, such as Snail, Twist and Zeb [34]. Recently, some studies have documented the involvement of EMT in HCC progression, including the loss of E-cadherin and the gain of Snail [35, 36]. To date, several deregulated miRNAs (miR-101, miR-124 and miR-148a) have been shown to regulate EMT in HCC [6, 7, 37]. In this study, we found that miR-449a is downregulated in HCC, especially in tissues with the PVTT. The inhibition of miR-449a in LO2 significantly promoted the transition of cells from an epithelial to a mesenchymal morphology. And the reintroduction of miR-449a into Huh7 and LM9 cells reverses EMT, as shown by the enhanced expression of the epithelial markers E-cadherin, α-catenin and β-catenin, and decreased expression of the mesenchymal markers fibronectin, N-cadherin and vimentin. So we identified miR-449a as a novel repressor on the EMT and metastasis of HCC cells.

It has been reported that FOS and Met has been shown to be overexpression in a variety of malignancies, including HCC [38–40]. And c-fos can induce epithelial–mesenchymal transition, which is associated with loss of cell polarity, in mammary epithelial cells [41, 42]. The cell surface receptor tyrosine kinase c-Met is upregulated in a variety of tumors [43, 44]. The increased c-Met signaling is associated with tumor growth and metastasis in human cancers [45]. Our results show that miR-449a inhibited the expression of FOS and Met by binding to 3’UTR and suppressed the downstream signaling. These observations are in accord with those of Skawran Britta and colleagues, who reported miR-449 binds c-Met mRNA to reduce its levels [27]. And Wang et al. also found that MicroRNA-449a is downregulated in non-small cell lung cancer and inhibits migration and invasion by targeting c-Met [20]. So the downregulation of miR-449a has been regarded as the mechanism which is responsible for the abnormal activation of c-Met.

It is known that the loss of E-cadherin expression has the important role in the EMT and metastasis of tumor cells. And the loss of E-cadherin expression may result from transcription suppression by Snail accumulation or by the promoter hypermethylation of E-cadherin gene [28, 46]. Here we showed that miR-449a repressed the activated phosphorylation of AKT and the inhibitory phosphorylation of GSK-3α/β, and consequently reduced the nuclear accumulation of Snail and enhanced E-cadherin expression. Interestingly, miR-449a may relieve E-cadherin from transcriptional repression by targeting Met/Snail signaling.

## Conclusions

In conclusion, our data suggest that downregulation of miR-449a plays an important role in HCC cell metastasis, and that miR-449a could be employed as a new prognostic marker and/or as an effective therapeutic target for HCC.

## Ethics approval

This study was approved by the Institute Research Medical Ethics Committee of Sun Yat-Sen Memorial Hospital, Guangzhou, China.

## Additional files

Additional file 1: Table S1. Sequences of real-time PCR primers for *Met* and *FOS*. (DOC 29 kb)
Additional file 2: Table S2. Univariate and Multivariate Analysis of Factors Associated with HCC patients’ Disease-Free Survival. (DOC 45 kb)
Additional file 3: Figure S1. Cell invasion was evaluated using a Matrigel invasion chamber. LM9 and Huh7 cells were infected by miR-control-lentivirus and miR-449a-lentivirus, respectively, or transfected with siFOS and siMET. All cells were subjected to a Matrigel invasion assay with fetal bovine serum as chemoattractant. Invasive cells were fixed and stained with crystal violet. The inserts were treated with 10 % acetic acid and the absorbance was measured. Both overexpression of miR-449a and knockdown of FOS and MET clearly inhibited the invasion of LM9 and Huh7 cells. Data are the means±SD of three independent experiments. **p* < 0.05, ***p* < 0.01. Scale bar: 100 mm. (TIFF 2444 kb)
Additional file 4: Figure S2. Enforced expression of miR-449a in HCC cell line inhibits the mRNA and protein levels of *Notch1*. (A) Enforced overexpression of miR-449a in Huh7 cells decreases endogenous levels of *Notch1* protein. Huh7 cells were infected with Mock, lent-miR-ctr or lenti-miR-449a for 72 hours. *Notch1* expression was assessed by Western blot. (B) MiR report constructs containing a wild-type and 2 mutated *Notch1* 3’UTRs were transfected into Huh7 cells, respectively. Relative repression of firefly luciferase expression was standardized to a transfection control. The reporter assays were performed 3 times with essentially identical results. (C) The mRNA levels of *Notch1* in Mock, lent-miR-ctr or lenti-miR-449a Huh7 cells examined by Real-time PCR. Lenti-miR-449a decreased the levels of *Notch1* mRNA in Huh7 cells. (D) Western blot assay showing protein levels of *Notch1* after the treatment of Mock, Anti-miRNC and anti-miR-449a in HepG2 cell line. Anti-miR-449a could increase *Notch1* expression in HepG2 cells. (TIFF 718 kb)
Additional file 5: Table S3. Correlations between the levels of miR-449a and the mRNA expression of *FOS* and *Met* in 77 HCCs. (DOC 35 kb)

## Abbreviations

miRNA: microRNA
HCC: Hepatocellular carcinoma
FOS: FBJ murine osteosarcoma viral oncogene homolog
MET: MET pro-oncogene, receptor tyrosine kinase
3-’UTR: 3**’**-untranslated region
EMT: Epithelial mesenchymal transfer
IF: Immunofluorescence
IHC: Immunohistochemistry

## References

[1] Polyak K, Weinberg RA (2009). Transitions between epithelial and mesenchymal states: acquisition of malignant and stem cell traits. Nat Rev Cancer.

[2] Kato M, Slack FJ (2008). MicroRNAs: small molecules with big roles - C. elegans to human cancer. Biology of the cell / under the auspices of the European Cell Biology Organization.

[3] Szabo G, Bala S (2013). MicroRNAs in liver disease. Nat Rev Gastroenterol Hepatol.

[4] Iorio MV, Croce CM (2012). MicroRNA dysregulation in cancer: diagnostics, monitoring and therapeutics. A comprehensive review. EMBO Mol Med.

[5] Oishi N, Kumar MR, Roessler S, Ji J, Forgues M, Budhu A (2012). Transcriptomic profiling reveals hepatic stem-like gene signatures and interplay of miR-200c and epithelial-mesenchymal transition in intrahepatic cholangiocarcinoma. Hepatology.

[6] Zheng F, Liao YJ, Cai MY, Liu YH, Liu TH, Chen SP (2012). The putative tumour suppressor microRNA-124 modulates hepatocellular carcinoma cell aggressiveness by repressing ROCK2 and EZH2. Gut.

[7] Zhang JP, Zeng C, Xu L, Gong J, Fang JH, Zhuang SM (2013). MicroRNA-148a suppresses the epithelial-mesenchymal transition and metastasis of hepatoma cells by targeting Met/Snail signaling. Oncogene.

[8] Rokavec M, Oner MG, Li H, Jackstadt R, Jiang L, Lodygin D (2014). IL-6R/STAT3/miR-34a feedback loop promotes EMT-mediated colorectal cancer invasion and metastasis. J Clin Invest.

[9] Colangelo T, Fucci A, Votino C, Sabatino L, Pancione M, Laudanna C (2013). MicroRNA-130b promotes tumor development and is associated with poor prognosis in colorectal cancer. Neoplasia.

[10] Papadimitriou E, Vasilaki E, Vorvis C, Iliopoulos D, Moustakas A, Kardassis D (2012). Differential regulation of the two RhoA-specific GEF isoforms Net1/Net1A by TGF-beta and miR-24: role in epithelial-to-mesenchymal transition. Oncogene.

[11] Wang SC, Lin XL, Li J, Zhang TT, Wang HY, Shi JW (2014). MicroRNA-122 Triggers Mesenchymal-Epithelial Transition and Suppresses Hepatocellular Carcinoma Cell Motility and Invasion by Targeting RhoA. PLoS One.

[12] Xia H, Ooi LL, Hui KM (2013). MicroRNA-216a/217-induced epithelial-mesenchymal transition targets PTEN and SMAD7 to promote drug resistance and recurrence of liver cancer. Hepatology.

[13] Zhang Q, He XJ, Ma LP, Li N, Yang J, Cheng YX (2011). Expression and significance of microRNAs in the p53 pathway in ovarian cancer cells and serous ovarian cancer tissues. Zhonghua zhong liu za zhi [Chinese journal of oncology].

[14] Ye W, Xue J, Zhang Q, Li F, Zhang W, Chen H (2014). MiR-449a functions as a tumor suppressor in endometrial cancer by targeting CDC25A. Oncol Rep.

[15] Ostling P, Leivonen SK, Aakula A, Kohonen P, Makela R, Hagman Z (2011). Systematic analysis of microRNAs targeting the androgen receptor in prostate cancer cells. Cancer Res.

[16] Chen H, Lin YW, Mao YQ, Wu J, Liu YF, Zheng XY (2012). MicroRNA-449a acts as a tumor suppressor in human bladder cancer through the regulation of pocket proteins. Cancer Lett.

[17] Martin A, Jones A, Bryar PJ, Mets M, Weinstein J, Zhang G (2013). MicroRNAs-449a and -449b exhibit tumor suppressive effects in retinoblastoma. Biochem Biophys Res Commun.

[18] Wei B, Song Y, Zhang Y, Hu M (2013). MicroRNA-449a functions as a tumor-suppressor in gastric adenocarcinoma by targeting Bcl-2. Oncology letters.

[19] Chen S, Dai Y, Zhang X, Jin D, Li X, Zhang Y (2014). Increased miR-449a expression in colorectal carcinoma tissues is inversely correlated with serum carcinoembryonic antigen. Oncology letters.

[20] Luo W, Huang B, Li Z, Li H, Sun L, Zhang Q (2013). MicroRNA-449a is downregulated in non-small cell lung cancer and inhibits migration and invasion by targeting c-Met. PLoS One.

[21] Hu J, Fang Y, Cao Y, Qin R, Chen Q (2014). MiR-449a Regulates proliferation and chemosensitivity to cisplatin by targeting cyclin D1 and BCL2 in SGC7901 cells. Dig Dis Sci.

[22] Liu YJ, Lin YF, Chen YF, Luo EC, Sher YP, Tsai MH (2013). MicroRNA-449a enhances radiosensitivity in CL1-0 lung adenocarcinoma cells. PLoS One.

[23] Noonan EJ, Place RF, Pookot D, Basak S, Whitson JM, Hirata H (2009). MiR-449a targets HDAC-1 and induces growth arrest in prostate cancer. Oncogene.

[24] Yang X, Feng M, Jiang X, Wu Z, Li Z, Aau M (2009). MiR-449a and miR-449b are direct transcriptional targets of E2F1 and negatively regulate pRb-E2F1 activity through a feedback loop by targeting CDK6 and CDC25A. Genes Dev.

[25] Ren XS, Yin MH, Zhang X, Wang Z, Feng SP, Wang GX (2014). Tumor-suppressive microRNA-449a induces growth arrest and senescence by targeting E2F3 in human lung cancer cells. Cancer Lett.

[26] Capuano M, Iaffaldano L, Tinto N, Montanaro D, Capobianco V, Izzo V (2011). MicroRNA-449a overexpression, reduced NOTCH1 signals and scarce goblet cells characterize the small intestine of celiac patients. PLoS One.

[27] Buurman R, Gurlevik E, Schaffer V, Eilers M, Sandbothe M, Kreipe H (2012). Histone deacetylases activate hepatocyte growth factor signaling by repressing microRNA-449 in hepatocellular carcinoma cells. Gastroenterology.

[28] Cano A, Perez-Moreno MA, Rodrigo I, Locascio A, Blanco MJ, del Barrio MG (2000). The transcription factor snail controls epithelial-mesenchymal transitions by repressing E-cadherin expression. Nat Cell Biol.

[29] Zhou BP, Deng J, Xia W, Xu J, Li YM, Gunduz M (2004). Dual regulation of Snail by GSK-3beta-mediated phosphorylation in control of epithelial-mesenchymal transition. Nat Cell Biol.

[30] Wang Z, Chen Z, Gao Y, Li N, Li B, Tan F (2011). DNA hypermethylation of microRNA-34b/c has prognostic value for stage non-small cell lung cancer. Cancer Biol Ther.

[31] Feng M, Yu Q (2010). MiR-449 regulates CDK-Rb-E2F1 through an auto-regulatory feedback circuit. Cell Cycle.

[32] Noonan EJ, Place RF, Basak S, Pookot D, Li LC (2010). MiR-449a causes Rb-dependent cell cycle arrest and senescence in prostate cancer cells. Oncotarget.

[33] Chen L, Chan TH, Yuan YF, Hu L, Huang J, Ma S (2010). CHD1L promotes hepatocellular carcinoma progression and metastasis in mice and is associated with these processes in human patients. J Clin Invest.

[34] Peinado H, Olmeda D, Cano A (2007). Snail, Zeb and bHLH factors in tumour progression: an alliance against the epithelial phenotype?. Nat Rev Cancer.

[35] Wei Y, Van Nhieu JT, Prigent S, Srivatanakul P, Tiollais P, Buendia MA (2002). Altered expression of E-cadherin in hepatocellular carcinoma: correlations with genetic alterations, beta-catenin expression, and clinical features. Hepatology.

[36] Yang MH, Chen CL, Chau GY, Chiou SH, Su CW, Chou TY (2009). Comprehensive analysis of the independent effect of twist and snail in promoting metastasis of hepatocellular carcinoma. Hepatology.

[37] Zheng F, Liao YJ, Cai MY, Liu TH, Chen SP, Wu PH (2015). Systemic delivery of microRNA-101 potently inhibits hepatocellular carcinoma in vivo by repressing multiple targets. PLoS Genet.

[38] Arbuthnot P, Kew M, Fitschen W (1991). C-fos and c-myc oncoprotein expression in human hepatocellular carcinomas. Anticancer Res.

[39] Yuen MF, Wu PC, Lai VC, Lau JY, Lai CL (2001). Expression of c-Myc, c-Fos, and c-jun in hepatocellular carcinoma. Cancer.

[40] Li S, Fu H, Wang Y, Tie Y, Xing R, Zhu J (2009). MicroRNA-101 regulates expression of the v-fos FBJ murine osteosarcoma viral oncogene homolog (FOS) oncogene in human hepatocellular carcinoma. Hepatology.

[41] Reichmann E, Schwarz H, Deiner EM, Leitner I, Eilers M, Berger J (1992). Activation of an inducible c-FosER fusion protein causes loss of epithelial polarity and triggers epithelial-fibroblastoid cell conversion. Cell.

[42] Fialka I, Schwarz H, Reichmann E, Oft M, Busslinger M, Beug H (1996). The estrogen-dependent c-JunER protein causes a reversible loss of mammary epithelial cell polarity involving a destabilization of adherens junctions. J Cell Biol.

[43] Gherardi E, Stoker M (1990). Hepatocytes and scatter factor. Nature.

[44] Peruzzi B, Bottaro DP (2006). Targeting the c-Met signaling pathway in cancer. Clinical cancer research : an official journal of the American Association for Cancer Research.

[45] You H, Ding W, Dang H, Jiang Y, Rountree CB (2011). C-Met represents a potential therapeutic target for personalized treatment in hepatocellular carcinoma. Hepatology.

[46] Lim SO, Gu JM, Kim MS, Kim HS, Park YN, Park CK (2008). Epigenetic changes induced by reactive oxygen species in hepatocellular carcinoma: methylation of the E-cadherin promoter. Gastroenterology.

