# Supplementary material for: MiR-449a suppresses the epithelial-mesenchymal transition and metastasis of hepatocellular carcinoma by multiple targets
Source: BMC Cancer. 2015 Oct 15;15:706. doi: 10.1186/s12885-015-1738-3 (PMC4608176; doi:10.1186/s12885-015-1738-3)
Supplement: Additional file 1: Table S1. — Sequences of real-time PCR primers for Met and FOS. (DOC 29 kb) [file 12885_2015_1738_MOESM1_ESM.doc]

| **Table S1 Sequences of real-time PCR primers for *Met* and *FOS*** | | | |
| --- | --- | --- | --- |
| **primer** | **sense** | **antisense** |  |
| Met | GGCTGGTGGCACTTTACTTA | CTTGTCTCTCGGTTGGCTA |  |
| FOS | ggggagccttcagagagagt | ttcttcctgggacggtgag |  |
| GAPDH | tcagtggtggacctgacctg | tgctgtagccaaattcgttg |  |
