# Supplementary material for: MiR-449a suppresses the epithelial-mesenchymal transition and metastasis of hepatocellular carcinoma by multiple targets
Source: BMC Cancer. 2015 Oct 15;15:706. doi: 10.1186/s12885-015-1738-3 (PMC4608176; doi:10.1186/s12885-015-1738-3)
Supplement: Additional file 2: Table S2. — Univariate and Multivariate Analysis of Factors Associated with HCC patients’ Disease-Free Survival. (DOC 45 kb) [file 12885_2015_1738_MOESM2_ESM.doc]

**Table S2** Univariate and Multivariate Analysis of Factors Associated with HCC patients’ Disease-Free Survival

| **Clinical Variable** | **Case number** | **HR (95% CI)** | ***P* value** |
| --- | --- | --- | --- |
| **Univariate analysis** |  |  |  |
| miR-449a (low versus high)* | 38/39 | 0.1 (0.1-0.3) | **0.000** |
| Sex (M versus F) | 67/10 | 1.2 (0.4-2.8) | 0.365 |
| Age (>50 versus ≤50 years) | 46/31 | 1.6 (0.4-2.1) | 0.495 |
| HBV (positive versus negative) | 67/10 | 1.4 (0.5-1.2) | 0.763 |
| Cirrhosis (yes versus no) | 55/22 | 1.1 (0.5-1.3) | 0.781 |
| AFP (>20 versus ≤20ng/ml) | 33/44 | 1.3 (0.8-2.1) | 0.086 |
| Tumor size (>5cm versus ≤5cm) | 34/43 | 2.8 (1.3-5.6) | **0.008** |
| Tumor nodule number (>1versus 1) | 30/47 | 2.3 (1.5-3.5) | **0.003** |
| TNM stage (I/II versus III/IV) | 30/47 | 1.6 (0.9-2.5) | **0.001** |
| Edmondson grade (I/II versus III/IV) | 53/24 | 1.5 (0.8-1.2) | 0.075 |
| Distant metastasis(M1/Mx) | 9/68 | 1.2(0.6-3.9) | **0.003** |
| **Multivariate analysis** |  |  |  |
| miR-449a (low versus high) | 38/39 | 0.2 (0.2-0.9) | **0.002** |
| TNM stage (I/II versus III/IV) | 30/47 | 1.3 (1.5-2.3) | **0.006** |
| Distant metastasis(M1/Mx) | 53/24 | 1.1 (0.7-1.4) | **0.014** |
| Analysis was conducted on 77 cases, Hazard ratios (95% confidence interval) and P values were calculated using univariate or multivariate Cox proportional hazard regression.  AFP, alpha-fetoprotein; HBV, hepatitis B virus, TNM, tumor-node-metastasis.  *The mature miR-449a level was examined by real-time qPCR and normalized to U6 level. The median value of all 77 samples was chosen as the cut-off point for separating miR-449a low tumors from miR-449a high tumors. | | | |
| Boldface *P* values indicate statistical significant. | | | |
