# Supplementary figures and images for: MiR-449a suppresses the epithelial-mesenchymal transition and metastasis of hepatocellular carcinoma by multiple targets
Source: BMC Cancer. 2015 Oct 15;15:706. doi: 10.1186/s12885-015-1738-3 (PMC4608176; doi:10.1186/s12885-015-1738-3)

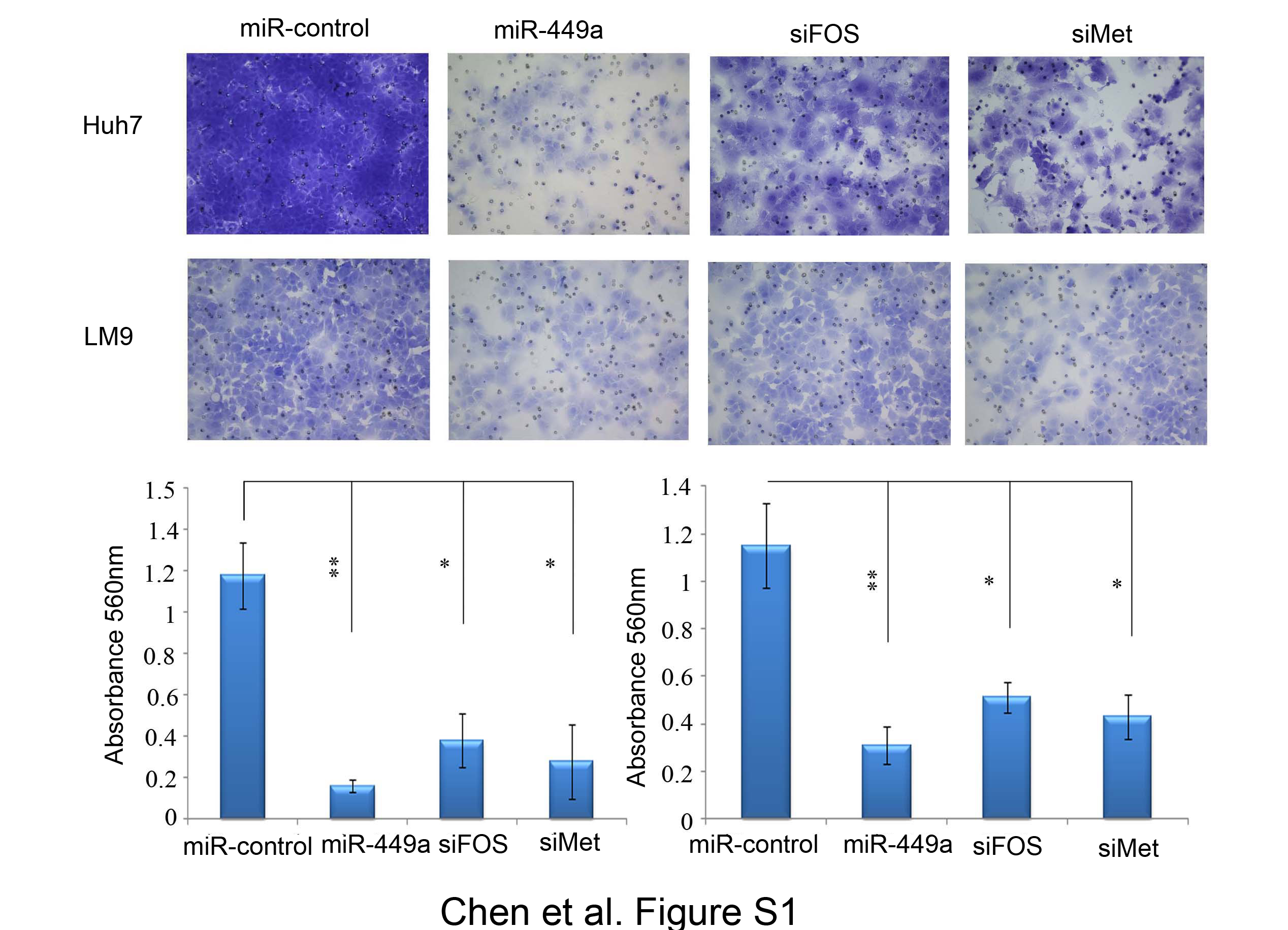

Supplement: Additional file 3: Figure S1. — Cell invasion was evaluated using a Matrigel invasion chamber. LM9 and Huh7 cells were infected by miR-control-lentivirus and miR-449a-lentivirus, respectively, or transfected with siFOS and siMET. All cells were subjected to a Matrigel invasion assay with fetal bovine serum as chemoattractant. Invasive cells were fixed and stained with crystal violet. The inserts were treated with 10 % acetic acid and the absorbance was measured. Both overexpression of miR-449a and knockdown of FOS and MET clearly inhibited the invasion of LM9 and Huh7 cells. Data are the means±SD of three independent experiments. *p < 0.05, **p < 0.01. Scale bar: 100 mm. (TIFF 2444 kb) [file 12885_2015_1738_MOESM3_ESM.tiff]

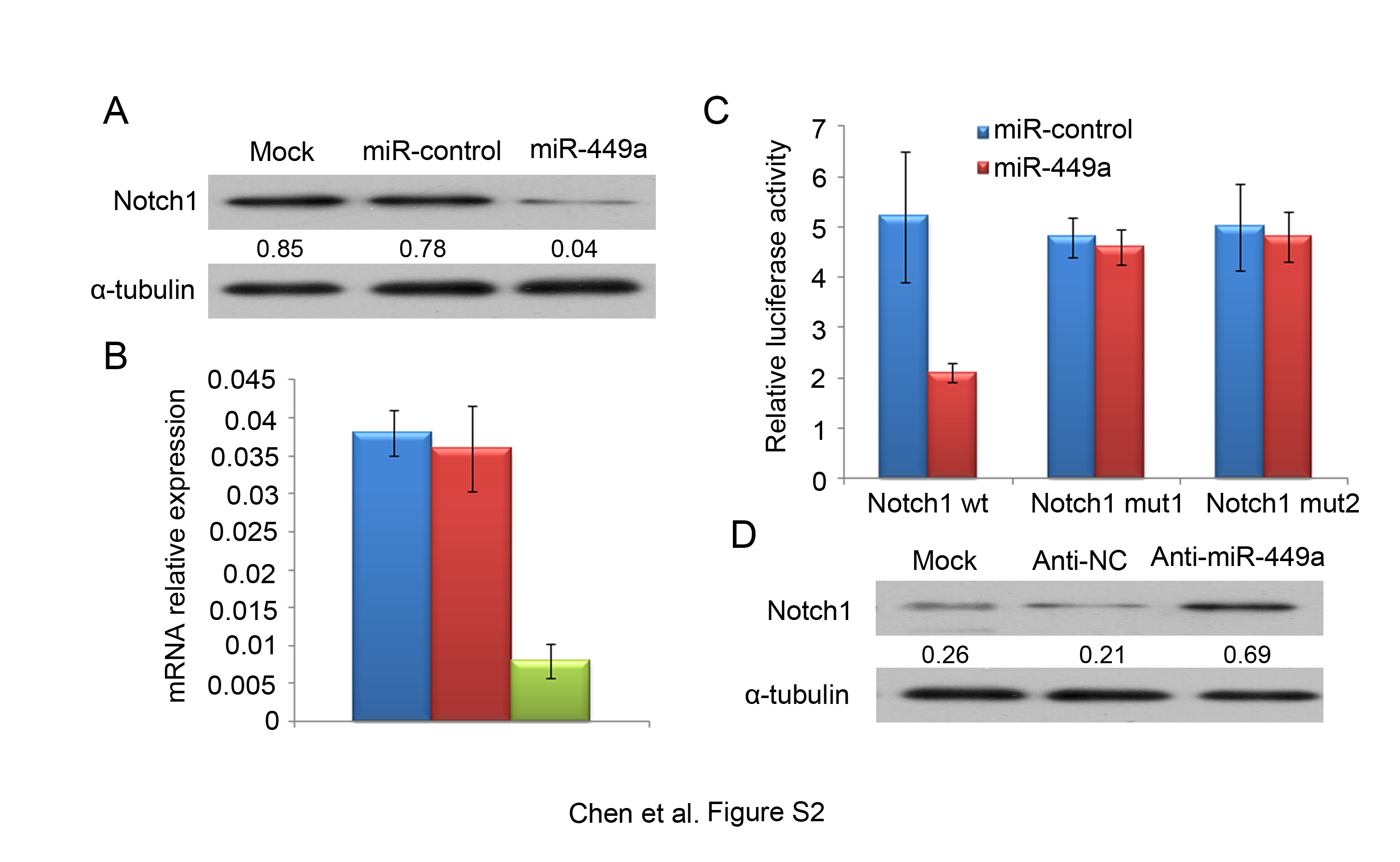

Supplement: Additional file 4: Figure S2. — Enforced expression of miR-449a in HCC cell line inhibits the mRNA and protein levels of Notch1. (A) Enforced overexpression of miR-449a in Huh7 cells decreases endogenous levels of Notch1 protein. Huh7 cells were infected with Mock, lent-miR-ctr or lenti-miR-449a for 72 hours. Notch1 expression was assessed by Western blot. (B) MiR report constructs containing a wild-type and 2 mutated Notch1 3’UTRs were transfected into Huh7 cells, respectively. Relative repression of firefly luciferase expression was standardized to a transfection control. The reporter assays were performed 3 times with essentially identical results. (C) The mRNA levels of Notch1 in Mock, lent-miR-ctr or lenti-miR-449a Huh7 cells examined by Real-time PCR. Lenti-miR-449a decreased the levels of Notch1 mRNA in Huh7 cells. (D) Western blot assay showing protein levels of Notch1 after the treatment of Mock, Anti-miRNC and anti-miR-449a in HepG2 cell line. Anti-miR-449a could increase Notch1 expression in HepG2 cells. (TIFF 718 kb) [file 12885_2015_1738_MOESM4_ESM.tiff]
