# Supplementary material for: MiR-449a suppresses the epithelial-mesenchymal transition and metastasis of hepatocellular carcinoma by multiple targets
Source: BMC Cancer. 2015 Oct 15;15:706. doi: 10.1186/s12885-015-1738-3 (PMC4608176; doi:10.1186/s12885-015-1738-3)
Supplement: Additional file 5: Table S3. — Correlations between the levels of miR-449a and the mRNA expression of FOS and Met in 77 HCCs. (DOC 35 kb) [file 12885_2015_1738_MOESM5_ESM.doc]

**Table S3** Correlations between the levels of miR-449a and the mRNA expression of *FOS* and *Met* in 77 HCCs

|  | | **MiR-449a levels** | | ***p* value^*^** |
| --- | --- | --- | --- | --- |
| **variable** | **Cases** | **Low** | **High** |
| FOS mRNA^†^ |  |  |  | 0.005 |
| High expression | 39 | 24 (61.5%) | 15 (38.5%) |  |
| Low expression | 38 | 15(39.4%) | 23(60.6%) |  |
| Met mRNA^†^ |  |  |  | 0.002 |
| High expression | 39 | 25(64.1%) | 14 (35.9%) |  |
| Low expression | 38 | 13 (34.2%) | 25 (65.8%) |  |

^*^Fishers exact test. ^†^The mRNA levels of FOS and Met were examined by real-time qPCR and normalized to GAPDH. The median value of all 77 samples was chosen as the cut-off point for separating FOS or Met mRNA low expression HCCs from that of high expression HCCs.
